# Supplementary material for: Factors of prescribing phage therapy among UK healthcare professionals: Evidence from conjoint experiment and interviews
Source: PLoS One. 2024 May 7;19(5):e0303056. doi: 10.1371/journal.pone.0303056 (PMC11075860; doi:10.1371/journal.pone.0303056)
Supplement: S1 File — (DOCX) [file pone.0303056.s001.docx]

**Supporting Information 1 – Interview Topic Guide**

1. Please briefly describe your role, or what your role was, as a prescriber?
2. In your experience, what are the factors which underpin prescribing decisions?
3. When deciding which medical treatment to prescribe a patient, which attributes of the treatment itself influence your decision the most?
4. What are your thoughts on antimicrobial resistance (AMR) seriousness?
5. How does AMR influence you/general practice when prescribing antibiotics? Have you seen a change in prescribing behaviour?
6. Have you felt pressure from patients to prescribe antibiotics?
7. What antibiotics alternative do you suggest to patients?
8. Are you aware of any potential antibiotic-alternative treatments other than phage therapy?
9. Had you heard of phage therapy before this interview?
10. Is there potential for phage therapy to be a routine treatment in healthcare in the future?
11. What challenges do you think implementing routine prescription of phage therapy may face?
12. What strategies do you believe are required to encourage public acceptance/uptake of phage therapy in the future?
13. If phage therapy was approved in the future, would you accept it as an alternative to antibiotics?
14. If phage therapy was approved in the future, would you feel comfortable prescribing it as an alternative to antibiotics?
