# Supplementary material for: Factors of prescribing phage therapy among UK healthcare professionals: Evidence from conjoint experiment and interviews
Source: PLoS One. 2024 May 7;19(5):e0303056. doi: 10.1371/journal.pone.0303056 (PMC11075860; doi:10.1371/journal.pone.0303056)
Supplement: S2 File — (DOCX) [file pone.0303056.s002.docx]

**Supporting Information 2 – Focus Group Topic Guide**

1. **Please briefly describe your role as a prescriber**

*Prompts:*

1. How long since you qualified/registered?
2. How long have you been in your current/most recent post?
3. Do you have a specialism?

1. **What do you think are the factors which underpin prescribing decisions in a general practice?**

*Prompts:*

1. Guidelines? e.g. National Institute for Health & Clinical Excellence (NICE), Quality & Outcomes Framework (QOF), Regional Medicines Optimisation Committee (RMOC) information or advice
2. Local influences? e.g. local/ area prescribing committee, incentive schemes, patient factors (population, specific patients)
3. Education and feedback? e.g. Local education programmes, informal learning (from colleagues), electronic data about prescribing, information from pharmaceutical industry

1. **What are your thoughts on antimicrobial resistance (AMR) in general?**

*Prompts:*

1. How would AMR influence you when prescribing antibiotics?
2. Do you think prescribing practices have changed over time as a result of antibiotic resistance?
3. Have you ever come across cases where antibiotics have not been effective in treating patients with probable bacterial infections, practical or through teaching? Any common infection types in particular?
4. How do you think development of antibiotic resistance can be reduced? (e.g. interventions, review of national guidelines, limit antibiotic marketing)

1. **Are you aware of any potential antibiotic-alternative treatments?**

*Prompts:*

1. Where/how do you find this information?
2. Have you heard of phage therapy before and, if yes, what do you understand it to be?
3. How do you think the potential of phage therapy compares to other antibiotic alternatives?

1. **Do you think phage therapy could be a viable alternative to antibiotics?**

*Prompts:*

1. If any, what are your concerns/hesitations with phage therapy being used as an antibiotic alternative?
2. What challenges do you think implementing routine prescription of phage therapy may face?
3. How do you think phage therapy could be implemented into general practice most successfully? (e.g. combination use with antibiotics, increase compassionate cases, phage proteins (lysins))
4. Are there any specific patient groups you believe could benefit from increasing phage therapy availability/access?

1. **Is there anything else you would like to add that has not been covered?**
