# Supplementary material for: Factors of prescribing phage therapy among UK healthcare professionals: Evidence from conjoint experiment and interviews
Source: PLoS One. 2024 May 7;19(5):e0303056. doi: 10.1371/journal.pone.0303056 (PMC11075860; doi:10.1371/journal.pone.0303056)
Supplement: S1 Table — (DOCX) [file pone.0303056.s003.docx]

**Supporting Information 3 – Sample Sociodemographic Characteristics**

| **Survey Question** | **Answer Categories** | **General Practitioners (%)** | **Health Professionals (%)** |
| --- | --- | --- | --- |
| **What is your ethnic group?**  **(n GP = 102; n HP = 101)** | Multiple ethnic groups  Asian / Asian British  White / White British  Black / African / Caribbean / Black British  Other ethnic group  Prefer not to say | 2.9  22.5  60.8  10.8  2  1 | 2  5.9  86.1  3  2  1 |
| **Where do you live?**  **(n GP = 102; n HP = 101)** | East of England  East Midlands  London  North East  North West  Northern Ireland  Scotland  South East  South West  Wales  West Midlands  Yorkshire & Humberside | 9.8  4.9  41.2  2  2.9  2  8.8  9.8  2.9  2.9  6.9  5.9 | 8.9  11.9  2  5.9  12.9  3  8.9  10.9  5.9  9.9  5.9  13.9 |
| **How would you describe yourself?**  **(n GP = 108; n HP = 100)** | Man  Woman  I do not identify as a man or woman  I prefer not to say | 63  37  0  0 | 5  91  1  3 |
| **Age**  **(n GP = 103; n HP = 100)** |  | Min=22  Max=68  Mean=35.7  Std=8.5 | Min=24  Max=75  Mean=44.5  Std=11.3 |
| **Which of the following best describes your employment status?**  **(n GP = 102; n HP = 101)** | Working full time  Working part time  Looking for work  Student  Retired  Not working nor looking for work  Other | 79.4  14.7  2.9  2.9  0  0  0 | 55.4  27.7  3  1  5.9  6.9  0 |
| **Please select from the below list the options that best describes the highest level of qualification you have attained to date.**  ***(multiple choice*)** | No Formal Qualification  Up to GCE O level, GSCE, School Certificate or equivalent qual.  A level, Higher Certificate or equivalent qual.  University degree or higher, or equivalent qual.  Postgr. medical training - The foundation programme  Postgr. medical training - Specialty and general practice training  Other  Don’t know  Prefer not to say | 0.8  5.3  3.1  15.3  18.3  42  0  0.8  0 | 0  3.9  21.4  61.2  3.9  4.9  0  0  1.9 |
| **Please select from the below list which council you are registered to practise with.**  **(*multiple choice*)** | General Medicine Council (GMC)  Health and Care Professions Council (HCPC)  Nursing & Midwifery Council (NMC)  General Dental Council (GDC)  Other  I am not registered  Don’t know  Prefer not to say | 40.5  30.5  7.6  5.3  3.1  1.5  0  0.8 | 6.8  12.6  34  2.9  9.7  28.2  0  1.9 |
