# Supplementary material for: Factors of prescribing phage therapy among UK healthcare professionals: Evidence from conjoint experiment and interviews
Source: PLoS One. 2024 May 7;19(5):e0303056. doi: 10.1371/journal.pone.0303056 (PMC11075860; doi:10.1371/journal.pone.0303056)
Supplement: S2 Table — (DOCX) [file pone.0303056.s004.docx]

**Supporting Information 4 – Coefficient Estimates for the Conjoint Experiment [AMCE Figures 2-4]**

| **Figure 2. Baseline Model: Discrete Choice AMCE** | | | | | | | |
| --- | --- | --- | --- | --- | --- | --- | --- |
| **feature** | **level** | **estimate** | **std.error** | **z** | **p** | **lower** | **upper** |
| Administration.route | Inhalation | 0.00 | NA | NA | NA | NA | NA |
| Administration.route | Intravenous | -0.05 | 0.03 | -1.82 | 0.07 | -0.11 | 0.00 |
| Administration.route | Oral | 0.00 | 0.03 | 0.03 | 0.98 | -0.05 | 0.05 |
| Patient.attitude.to.treatment | Extreme hesitance | 0.00 | NA | NA | NA | NA | NA |
| Patient.attitude.to.treatment | No hesitance | 0.05 | 0.03 | 1.97 | 0.05 | 0.00 | 0.11 |
| Patient.attitude.to.treatment | Some hesitance | 0.03 | 0.03 | 0.99 | 0.32 | -0.03 | 0.08 |
| Severity.of.infection | Acute | 0.00 | NA | NA | NA | NA | NA |
| Severity.of.infection | Chronic | 0.01 | 0.02 | 0.25 | 0.80 | -0.04 | 0.05 |
| Side.effect.rate | 1% | 0.00 | NA | NA | NA | NA | NA |
| Side.effect.rate | 10% | -0.07 | 0.03 | -2.15 | 0.03 | -0.13 | -0.01 |
| Side.effect.rate | 20% | -0.20 | 0.03 | -6.58 | 0.00 | -0.26 | -0.14 |
| Side.effect.rate | 5% | -0.06 | 0.03 | -1.77 | 0.08 | -0.12 | 0.01 |
| Success.rate | 20% | 0.00 | NA | NA | NA | NA | NA |
| Success.rate | 50% | 0.16 | 0.03 | 6.24 | 0.00 | 0.11 | 0.21 |
| Success.rate | 80% | 0.29 | 0.03 | 9.27 | 0.00 | 0.23 | 0.35 |
| Type.of.treatment | Antibiotics only | 0.00 | NA | NA | NA | NA | NA |
| Type.of.treatment | One phage only | 0.02 | 0.03 | 0.57 | 0.57 | -0.04 | 0.08 |
| Type.of.treatment | Phage cocktail | 0.02 | 0.03 | 0.60 | 0.55 | -0.04 | 0.08 |
| Type.of.treatment | Phage plus antibiotic | 0.01 | 0.03 | 0.23 | 0.82 | -0.05 | 0.07 |

Note: DCE AMCES estimates based on “cj” command in cregg. N=187 after respondents who failed the attention check or had missing data on the DCE are removed.

| **Figure 3. Ranked Evaluations of Alternative Treatments** | | | | | | | |
| --- | --- | --- | --- | --- | --- | --- | --- |
| **feature** | **level** | **estimate** | **std.error** | **z** | **p** | **lower** | **upper** |
| Administration.route | Inhalation | 0.00 | NA | NA | NA | NA | NA |
| Administration.route | Intravenous | 0.04 | 0.12 | 0.30 | 0.76 | -0.20 | 0.28 |
| Administration.route | Oral | 0.15 | 0.11 | 1.29 | 0.20 | -0.08 | 0.37 |
| Patient.attitude.to.treatment | Extreme hesitance | 0.00 | NA | NA | NA | NA | NA |
| Patient.attitude.to.treatment | No hesitance | 0.27 | 0.12 | 2.26 | 0.02 | 0.04 | 0.50 |
| Patient.attitude.to.treatment | Some hesitance | 0.11 | 0.12 | 0.87 | 0.38 | -0.13 | 0.34 |
| Severity.of.infection | Acute | 0.00 | NA | NA | NA | NA | NA |
| Severity.of.infection | Chronic | 0.17 | 0.10 | 1.66 | 0.10 | -0.03 | 0.38 |
| Side.effect.rate | 1% | 0.00 | NA | NA | NA | NA | NA |
| Side.effect.rate | 10% | -0.42 | 0.16 | -2.54 | 0.01 | -0.73 | -0.10 |
| Side.effect.rate | 20% | -0.78 | 0.16 | -4.95 | 0.00 | -1.09 | -0.47 |
| Side.effect.rate | 5% | -0.20 | 0.16 | -1.23 | 0.22 | -0.52 | 0.12 |
| Success.rate | 20% | 0.00 | NA | NA | NA | NA | NA |
| Success.rate | 50% | 0.62 | 0.13 | 4.81 | 0.00 | 0.37 | 0.87 |
| Success.rate | 80% | 1.16 | 0.15 | 7.70 | 0.00 | 0.86 | 1.45 |
| Type.of.treatment | Antibiotics only | 0.00 | NA | NA | NA | NA | NA |
| Type.of.treatment | One phage only | -0.12 | 0.16 | -0.79 | 0.43 | -0.43 | 0.18 |
| Type.of.treatment | Phage cocktail | 0.08 | 0.14 | 0.55 | 0.58 | -0.20 | 0.36 |
| Type.of.treatment | Phage plus antibiotic | -0.06 | 0.14 | -0.42 | 0.68 | -0.34 | 0.22 |

Note: DCE AMCES estimates based on “cj” command in cregg for evaluation on 10pt scale of alternative treatments.. N=187 after respondents who failed the attention check or had missing data on the DCE are removed.

| **Figure 4. AMCE estimates by Subgroups in the Sample: GPs and Health Professionals** | | | | | | | |
| --- | --- | --- | --- | --- | --- | --- | --- |
| **feature** | **level** | **estimate** | **std.error** | **z** | **p** | **lower** | **upper** |
| **Health Professionals** |  |  |  |  |  |  |  |
| Administration.route | Inhalation | 0.00 | NA | NA | NA | NA | NA |
| Administration.route | Intraveneous | -0.05 | 0.04 | -1.19 | 0.24 | -0.13 | 0.03 |
| Administration.route | Oral | 0.07 | 0.04 | 1.81 | 0.07 | -0.01 | 0.14 |
| Patient.attitude.to.treatment | Extreme hesitance | 0.00 | NA | NA | NA | NA | NA |
| Patient.attitude.to.treatment | No hesitance | 0.07 | 0.04 | 1.80 | 0.07 | -0.01 | 0.14 |
| Patient.attitude.to.treatment | Some hesitance | 0.04 | 0.04 | 1.14 | 0.26 | -0.03 | 0.11 |
| Severity.of.infection | Acute | 0.00 | NA | NA | NA | NA | NA |
| Severity.of.infection | Chronic | 0.02 | 0.03 | 0.80 | 0.42 | -0.03 | 0.08 |
| Side.effect.rate | 1% | 0.00 | NA | NA | NA | NA | NA |
| Side.effect.rate | 10% | -0.12 | 0.04 | -2.74 | 0.01 | -0.20 | -0.03 |
| Side.effect.rate | 20% | -0.28 | 0.04 | -6.86 | 0.00 | -0.36 | -0.20 |
| Side.effect.rate | 5% | -0.07 | 0.04 | -1.64 | 0.10 | -0.16 | 0.01 |
| Success.rate | 20% | 0.00 | NA | NA | NA | NA | NA |
| Success.rate | 50% | 0.26 | 0.03 | 7.76 | 0.00 | 0.19 | 0.32 |
| Success.rate | 80% | 0.45 | 0.04 | 11.56 | 0.00 | 0.38 | 0.53 |
| Type.of.treatment | Antibiotics only | 0.00 | NA | NA | NA | NA | NA |
| Type.of.treatment | One phage only | 0.07 | 0.04 | 1.62 | 0.11 | -0.01 | 0.15 |
| Type.of.treatment | Phage cocktail | 0.05 | 0.04 | 1.37 | 0.17 | -0.02 | 0.13 |
| Type.of.treatment | Phage plus antibiotic | 0.01 | 0.03 | 0.17 | 0.86 | -0.06 | 0.07 |
| **GPs** |  |  |  |  |  |  |  |
| Administration.route | Inhalation | 0.00 | NA | NA | NA | NA | NA |
| Administration.route | Intraveneous | -0.07 | 0.04 | -1.90 | 0.06 | -0.15 | 0.00 |
| Administration.route | Oral | -0.06 | 0.04 | -1.42 | 0.16 | -0.14 | 0.02 |
| Patient.attitude.to.treatment | Extreme hesitance | 0.00 | NA | NA | NA | NA | NA |
| Patient.attitude.to.treatment | No hesitance | 0.05 | 0.04 | 1.32 | 0.19 | -0.03 | 0.14 |
| Patient.attitude.to.treatment | Some hesitance | 0.04 | 0.05 | 0.88 | 0.38 | -0.05 | 0.13 |
| Severity.of.infection | Acute | 0.00 | NA | NA | NA | NA | NA |
| Severity.of.infection | Chronic | -0.01 | 0.04 | -0.28 | 0.78 | -0.08 | 0.06 |
| Side.effect.rate | 1% | 0.00 | NA | NA | NA | NA | NA |
| Side.effect.rate | 10% | 0.00 | 0.05 | -0.09 | 0.93 | -0.10 | 0.09 |
| Side.effect.rate | 20% | -0.11 | 0.05 | -2.43 | 0.02 | -0.20 | -0.02 |
| Side.effect.rate | 5% | -0.05 | 0.05 | -1.11 | 0.27 | -0.15 | 0.04 |
| Success.rate | 20% | 0.00 | NA | NA | NA | NA | NA |
| Success.rate | 50% | 0.06 | 0.04 | 1.56 | 0.12 | -0.02 | 0.13 |
| Success.rate | 80% | 0.16 | 0.05 | 3.54 | 0.00 | 0.07 | 0.26 |
| Type.of.treatment | Antibiotics only | 0.00 | NA | NA | NA | NA | NA |
| Type.of.treatment | One phage only | -0.04 | 0.05 | -0.82 | 0.41 | -0.14 | 0.06 |
| Type.of.treatment | Phage cocktail | -0.02 | 0.05 | -0.43 | 0.67 | -0.12 | 0.08 |
| Type.of.treatment | Phage plus antibiotic | 0.00 | 0.05 | 0.00 | 1.00 | -0.10 | 0.10 |

Note: DCE AMCES estimates based on “cj” command in cregg for evaluation on 10pt scale of alternative treatments.. N=187 after respondents who failed the attention check or had missing data on the DCE are removed.
